# Investigating the role of contextual cues and interhemispheric inhibitory mechanisms in response-selective stopping: a TMS study

**DOI:** 10.3758/s13415-022-01047-3

**Published:** 2022-11-16

**Authors:** Rohan Puri, Rebecca J. St George, Mark R. Hinder

**Affiliations:** 1 Sensorimotor Neuroscience and Ageing Research Group, Private Bag 30, School of Psychological Sciences, College of Health and Medicine, University of Tasmania, Hobart, TAS 7001 Australia; 2 Wicking Dementia Research and Education Centre, College of Health and Medicine, University of Tasmania, Hobart, Australia

**Keywords:** Inhibitory control, Response inhibition, Selective stopping, Transcranial magnetic stimulation, Interhemispheric inhibition, Bayes

## Abstract

Response-selective stopping requires cancellation of only one component of a multicomponent action. While research has investigated how delays to the continuing action components (“stopping interference”) can be attenuated by way of contextual cues of the specific stopping demands (“foreknowledge”), little is known of the underlying neural mechanisms. Twenty-seven, healthy, young adults undertook a multicomponent stop-signal task. For two thirds of trials, participants responded to an imperative (go) stimulus (IS) with simultaneous button presses using their left and right index fingers. For the remaining one third of trials, the IS was followed by a stop-signal requiring cancellation of *only* the left, *or* right, response. To manipulate foreknowledge of stopping demands, a cue preceded the IS that informed participants which hand *might* be required to stop (proactive) or provided no such information (reactive). Transcranial magnetic stimulation (TMS) assessed corticospinal excitability (CSE) as well as short- and long-interval interhemispheric inhibition (SIHI, LIHI) between the primary motor cortices. Proactive cues reduced, but did not eliminate, stopping interference relative to the reactive condition. Relative to TMS measures at cue onset, decreases in CSE (both hands and both cue conditions) and LIHI (both hands, proactive condition only) were observed during movement preparation. During movement cancellation, LIHI reduction in the continuing hand was greater than that in the stopping hand and greater than LIHI reductions in both hands during execution of multicomponent responses. Our results indicate that foreknowledge attenuates stopping interference and provide evidence for a novel role of LIHI, mediated via prefrontal regions, in facilitating continuing action components.

## Introduction

Response inhibition—the ability to cancel initiated actions—is a fundamental cognitive processes that is critical for behaving in a goal-directed manner (for reviews, see Duque et al., 2017; Nikitenko et al., 2020). Numerous day-to-day activities require response inhibition, with behavioural stopping occurring either nonselectively or selectively (Bissett and Logan, 2014). Nonselective stopping entails a termination of *all* movement components, whereas selective stopping involves stopping in a *stimulus-*selective or *response-*selective (as per Wadsley et al., 2022; termed “*motor*-selective” by Bissett and Logan, 2014) manner. For example, consider turning a corner while riding a bicycle when you are suddenly presented with a pothole. Based on the size of the pothole, you may implement stimulus-selective stopping. That is, you may brake to a stop if it is a big deep pothole or continue riding if it is a small shallow pothole. Based on your riding experience, you may implement nonselective stopping (i.e., a beginner may stop riding completely) or response-selective stopping (i.e., a more experienced rider may stop pedalling while steering away from the pothole).

In a laboratory setting, response-selective stopping is assessed via *multicomponent* variants of traditional response inhibition paradigms, such as the stop-signal task (SST; Lappin and Eriksen, 1966) and the anticipated response inhibition task (ARI; Slater-Hammel, 1960). In these traditional tasks, on a minority of trials, a stop-signal is presented, which requires participants to try and inhibit the default, single effector, go response (which is usually a button press in the SST and a time-locked anticipated button press or release in the ARI task). Using the probability of successfully stopping at various temporal delays between the go- and stop-signals, and the average go reaction-time (RT), the speed (or efficiency) of an individual’s ability to inhibit movement, termed stop-signal reaction-time (SSRT), can be calculated (for a review, see Verbruggen et al., 2019). In multicomponent variants of these tasks, first implemented in the ARI task by Coxon et al. (2007), instead of a single effector go response, the go response is a multieffector response. For example, on a single trial, if a leftward pointing arrow requires a left index finger button press in the traditional version of the SST, on a multicomponent version a left and rightward pointing arrow would require a bimanual simultaneous left and right index button press. This multieffector go response allows response-selective stopping to be assessed, as a stop-signal which is specific to only one stimulus (e.g., left arrow) requires participants to stop one subcomponent of the multieffector response (e.g., left index finger) while continuing to respond, as efficiently as possible, with the other subcomponent (e.g., right index finger).

One of the most prominent findings of response-selective stopping tasks is the “stopping interference” effect, whereby successfully stopping one effector leads to a RT delay (relative to go responses) in the continuing effector (i.e., the act of stopping one effector *interferes* with the speed of the continuing effector). This effect is reliably produced in response-selective SST (Cai et al., 2011; Claffey et al., 2010; Majid et al., 2012; Raud and Huster, 2017) as well as ARI (Cirillo et al., 2017; Cowie et al., 2016; Coxon et al., 2007; MacDonald et al., 2012; Wadsley et al., 2019) paradigms (for a comprehensive reference list, see Wadsley et al., 2022). One method shown to attenuate stopping interference is to provide participants with foreknowledge, usually via a warning cue before the imperative (go) cue, of which effector would need to be stopped if a stop-signal were to be presented (Aron and Verbruggen, 2008). For example, if a stop-signal required stopping of the left index finger (and consequently, continuing to respond with the right index finger), then a warning cue may read “Maybe stop left” (vs. an uninformative fixation cross). By utilizing this foreknowledge, participants can *proactively* engage inhibitory processes (vs. *reactively* engaging inhibitory processes when no foreknowledge is provided)^1^ to enable successful stopping and quicker responding of the corresponding effectors (i.e., reduced stopping interference effects).

Neuroimaging research has implicated two pathways in the cortico-subcortical network involved in response-selective stopping. Specifically, a “hyperdirect” pathway involving connections between the subthalamic nucleus (STN) and cortical areas of the right inferior frontal cortex and presupplementary motor area, and an “indirect” pathway involving connections between the aforementioned cortical areas and the STN but via the striatum (for a review, see Aron et al., 2016). Even though both pathways are implicated in response-selective stopping, there is suggestion of a greater engagement of the indirect pathway in proactive response-selective stopping (Cai et al., 2012; Coxon et al., 2009; Leunissen et al., 2016; Majid et al., 2013). Focussing on the primary motor cortex (M1), as the final target of these stopping networks (i.e., the cortical area responsible for the release, or withholding, of the motor command), transcranial magnetic stimulation (TMS) studies have provided valuable insights by quantifying the amplitude of the motor-evoked potential (MEP) as a measure of corticospinal excitability (CSE). Briefly, TMS is a noninvasive technique that when applied over M1 elicits descending volleys in corticospinal neurons that synapse onto spinal motoneurons innervating peripheral muscles, such as those in the hand (for a review, see Bestmann & Krakauer, 2015). Moreover, sophisticated TMS protocols can target specific intra- and inter-cortical circuits that provide novel insights into various behavioural aspects of movement preparation, execution, and cancellation (for a review, see Duque et al., 2017). During reactive response-selective stopping, reduced CSE (compared with rest or a task-relevant baseline) is observed not only in the stopped effector but also in the continuing effector, suggesting the recruitment of nonselective inhibitory neurophysiological processes (Cowie et al., 2016; MacDonald et al., 2014). In contrast, during proactive response-selective stopping, some selectivity is observed as reduced CSE in only the effector that may have to cancel its response (Cai et al., 2011), with a release of intracortical inhibition observed in the effector that is not cued to stop, and thus will continue its response (Cirillo et al., 2017).

Given the bimanual nature of most response-selective stopping paradigms, surprisingly little is known about *interactions* between the left and right M1s. Using dual-coil TMS at different interstimulus intervals, it is possible to investigate direct (transcallosal) and indirect (via premotor areas) interhemispheric connections (Ni et al. 2009) between primary motor cortices during both movement preparation (Hinder et al. 2018) and action cancellation (Puri et al., 2018). Recently, MacDonald et al. (2021) suggested a role of direct interhemispheric M1 circuits not only in nonselective stopping but also in facilitating selective initiation of the continuing effector during reactive response-selective stopping (but see Puri, 2021 for statistical concerns). To the best of our knowledge, no study to date has examined the role of direct and indirect interhemispheric M1 circuits in reactive and proactive response-selective stopping. Thus, the overall goal of the current study was to gain a deeper understanding of the role of direct and indirect interhemispheric connections between primary motor cortices in subserving reactive and proactive response-selective stopping. Based on previous literature and the complexity of response-selective stopping, we expected indirect interhemispheric M1 circuits (mediated by premotor regions and influenced by top-down processing) to play a prominent role, especially in a proactive context.

## Materials and methods

### Participants

Twenty-seven, healthy, young adults (mean age = 26.4 years; standard deviation [SD] = 5.3 years; range = 19-41 years; all self-declaring right-handed dominance) were recruited from the university and broader community. A medical history questionnaire assessed contraindications to TMS, and all participants were free of any known neuromuscular or neurological dysfunction. Participants provided written, informed consent before commencing the study, which was approved by the Tasmanian Human Research Ethics Committee Network and conducted in accordance with the Declaration of Helsinki.

### Experimental procedure

**Fig. 1:**
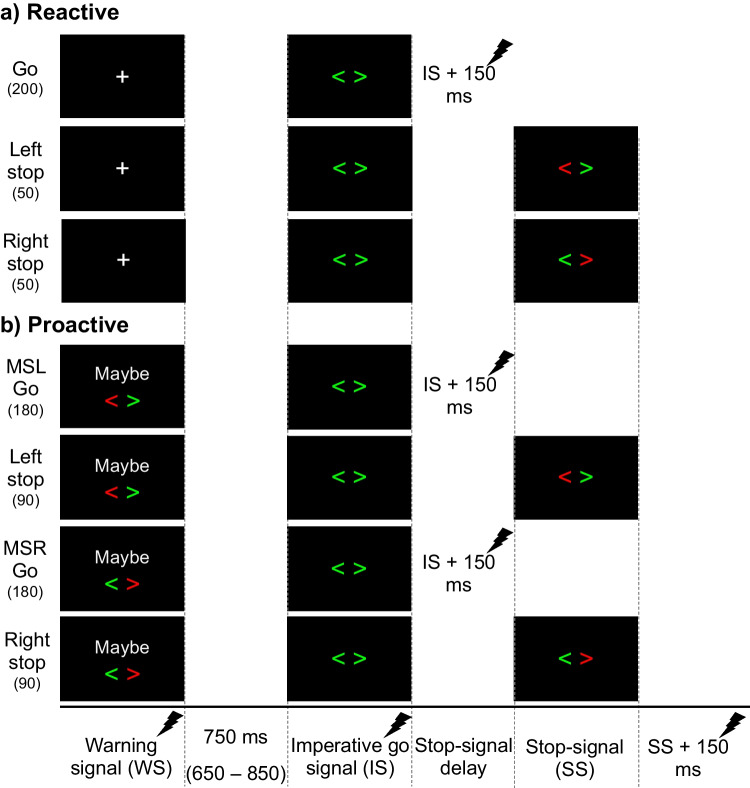
**Experimental trials. **Participants were visually presented, on a computer screen, a multicomponent stop-signal task, comprising of go trials (2/3^rd^ of all trials) and stop trials (1/3^rd^ of all trials). On go trials, an imperative signal (IS) of green arrows pointing to the left (“<”) and right (“>”) required participants to make a bimanual response using their left and right index fingers. On response-selective stop trials, after a stop-signal delay, either the left (*left stop*) or right (*right stop*) green IS arrow turned red, requiring participants to cancel their left, or right, index finger, respectively, while continuing to press the other button. In both go and stop trials, the IS was preceded by a warning signal (WS) that was either uninformative (fixation cross: +), probing **a)** reactive response-selective stopping, or informative (*Maybe Stop Left*: MSL; *Maybe Stop Right*: MSR), probing **b)** proactive response-selective stopping, about potential upcoming stopping demands. Numbers, in parentheses, below trial types indicate total trial numbers and filled lightning bolts indicate one of the four possible time-points (WS, IS, IS150, and SS150) at which TMS was administered on a single trial, noting that SS150 was only possible on stop trials

To investigate reactive and proactive response-selective stopping processes, a multicomponent stop-signal task, described in detail below and illustrated in Fig. 1, was performed in a single experimental session lasting between 1.5–2 h.

Participants were seated with their forearms pronated and resting on a table. Each index finger was positioned on a button of a USB response box (The Black Box ToolKit, Sheffield, UK). Visual stimuli were presented on a computer screen which necessitated specific responses. On two-thirds of all trials (“go” trials), an imperative signal (IS) consisting of two green arrows pointing to the left and to the right (“< >”) was presented for 750 ms. Participants were required to respond bimanually, by pressing the left and right buttons, simultaneously, as quickly as possible. Upon completion of a response (or a maximum response window of 2,000 ms in which no response was registered), a 500-ms blank screen was followed by trial feedback (displayed for 750 ms). Feedback constituted one of the following: a) Reaction time (RT), in seconds, if both button presses were registered as synchronous (defined as <50 ms between the two button presses); b) “Press simultaneously” if the two button presses were registered as asynchronous (i.e., ≥50 ms between the two button presses), classified as an error; c) “Respond quicker” if the RT of synchronous button presses was more than 250 ms slower than the average RT on go trials during the “go only” block (see below); d) “Incomplete” if only one of the two button presses was recorded, classified as an error; or e) “Missed” if no button press was recorded. The “respond quicker” feedback was implemented to discourage strategic slowing on go trials that is common in stop-signal tasks (Verbruggen et al., 2019).

On the remaining one-third of trials (“stop” trials), participants were presented with the same aforementioned IS. However, following a stop-signal delay (SSD; initial SSD = 200 ms) one of the green arrows turned red, requiring participants to selectively cancel the button press of the corresponding finger while continuing to respond with the other finger. For example, if the left green arrow turned red, it required participants to cancel the button press with the left index finger whilst continuing to press the button with the right index finger (“left stop”) and vice-versa (“right stop”). Following a 500-ms blank screen, feedback was displayed for 750 ms, constituting one of the following: a) “Failed to stop” if a bimanual button press was registered; b) RT, in seconds, of the continuing finger if the finger required to stop, cancelled the button press successfully (“successful stop”); c) RT, in seconds, if, on rare occasions the bimanual response was made before the stop-signal was displayed; d) “Stopped wrong hand” if the hand required to stop, responded, and the hand required to respond, stopped; and e) “Missed” if no response was recorded. SSDs were staircased, such that after failed stop trials SSD decreased by 50 ms (with a lower SSD limit of 50 ms) and increased by 50 ms after successful stops. This procedure was designed to yield ~50% successful stopping in both left stop (left hand) and right stop (right hand) trials (staircased independently for each hand and condition, see subsequent paragraph and Fig. 1 for different conditions).

To investigate different response-selective stopping processes, a 750-ms warning signal (WS) preceded the IS by a mean duration of 750 ms (blank screen period between 650 and 850 ms with values sampled from a truncated exponential distribution to limit anticipatory responses). Specifically, the informativeness of the WS was manipulated such that it provided no information about possible upcoming stopping demands (reactive condition: WS was a fixation cross, “+”) or valid information about possible upcoming stopping demands (proactive condition: WS indicated stopping of either the left - “Maybe Stop Left”, MSL - or right - “Maybe Stop Right”, MSR - hand might be required).

Participants began the experimental session with a block of 30 “go only” trials (i.e., no stop trials) to not only emphasize the go component of the task but also to enable the determination of a quantitative threshold to discourage waiting and strategic slowing in the main task blocks (Verbruggen et al., 2019). Following this, participants underwent the reactive and proactive blocks, counterbalanced across participants. Specifically, after a practice reactive block (30 trials comprising of 20 bimanual go trials, 5 left stop trials, and 5 right stop trials), participants undertook the main reactive blocks (5 blocks of 60 trials each resulting in 200 bimanual go trials, 50 left stop trials, and 50 right stop trials). Similarly, after a practice proactive block of 30 trials comprising 15 trials with a MSL cue (10 bimanual go and 5 left stop) and 15 trials with a MSR cue (10 bimanual go and 5 right stop), participants undertook the main proactive blocks (10 blocks of 54 trials each resulting in 270 trials each with MSL and MSR cues, of which 180 were go trials and 90 were stop trials). The inter-trial interval was set to 1,000 ms, and all experimental stimuli were presented using PsychoPy v1.84.2 (Peirce et al., 2019). Last, to minimize fatigue between blocks, participants were provided with self-timed breaks (minimum of 30 s) between blocks.

On the majority of trials (250 of 300 reactive trials and 480 of 540 proactive trials), TMS—either to measure corticospinal excitability (CSE), short-interval interhemispheric inhibition (SIHI), or long-interval interhemispheric inhibition (LIHI) (see “Electromyography and transcranial magnetic stimulation”﻿)—was delivered at one of four timepoints. TMS at the onset of the **WS** provided a task-relevant baseline of CSE and IHI; TMS at the onset of the **IS** allowed us to infer movement preparation effects on CSE and IHI (which could be influenced by the nature of the WS); TMS 150 ms *after* the IS onset (**IS150**) was used to infer movement execution related changes to M1 excitability and connectivity. Finally, on stop trials, TMS 150 ms after the onset of the stop-signal (**SS150**) was used to infer movement cancellation related changes in response to the stop-signal. The rationale for choosing 150 ms post-SS (SS150) was driven by recent research in stop-signal tasks reporting inhibition at this time-point (Raud and Huster, 2017; Raud et al., 2020) with IS150 chosen to maintain temporal consistency between go and stop trials. For the SIHI and LIHI conditions, the test TMS pulse was administered at the exact time points, with the conditioning pulse administered either 10 or 40 ms prior, respectively, to these predefined timepoints. In the reactive condition, 10 stimulations for each TMS condition were administered at WS, IS, and IS150 on go trials to infer CSE from both M1s (CSE_L_ and CSE_R_), SIHI from left to right M1 (SIHI_LR_), LIHI from left to right M1 (LIHI_LR_), SIHI from right to left M1 (SIHI_RL_), and LIHI from right to left M1 (LIHI_RL_). Twenty TMS trials were administered at SS150 on stop trials, half each for left and right stop trials, to infer CSE (CSE_L_ and CSE_R_), SIHI_LR_, LIHI_LR,_ SIHI_RL,_ and LIHI_RL_. In the proactive blocks, for the MSL and MSR cues separately, 10 trials each were administered at WS, IS, IS150 on go trials, and SS150 on stop trials to infer CSE (CSE_L_ and CSE_R_), SIHI_LR_, LIHI_LR_, SIHI_RL_, and LIHI_RL_. In addition, again for MSL and MSR cues separately, 20 TMS trials were administered at IS and IS150 on *stop* trials to infer if CSE at those timepoints determined stopping success. Both the type (CSE, SIHI, or LIHI) and timepoint (WS, IS, IS150, or SS150) of TMS administration throughout the experiment was pseudorandomized (“pseudo” aspect relating to ensuring that an equal number of each TMS type and timepoint were administered across all relevant blocks of trials). Last, 50 TMS trials (10 each of CSE: half each of CSE_L_ and CSE_R_, SIHI_LR_, LIHI_LR_, SIHI_RL_, and LIHI_RL_ types) were administered before the experimental conditions to ensure stimulation parameters were adequate to capture CSE, SIHI, and LIHI (see “Electromyography and transcranial magnetic stimulation”).

### Electromyography and transcranial magnetic stimulation

Electromyographic (EMG) surface electrodes (Ag/AgCl) were placed in a belly tendon montage over the left and right first dorsal interosseus (FDI) muscle, with the ground electrode placed ipsilaterally over the radial styloid process. Signals were sampled at 2,000 Hz, amplified with a gain of 1,000, band-pass filtered (20–1,000 Hz), and stored for offline analysis using a 16-bit AD system (CED Power1401 and CED 1902, Cambridge, UK). Using a computer monitor, participants’ online EMG activity was monitored by the experimenter to ensure muscle relaxation, and when necessary, participants were reminded to keep their hands relaxed before and after the voluntary button presses.

To assess CSE in both M1s (CSE_L_ and CSE_R_) and IHI from the left to right M1 as well as from the right to left M1 (SIHI_LR_, LIHI_LR_, SIHI_RL_, and LIHI_RL_), dual-coil TMS protocols were utilized. Two “branding iron” style figure-of-eight coils (external diameter of each wing ~70 mm), connected to two Magstim 200^2^ stimulators (Magstim Company, Dyfed, UK), were used to deliver TMS to the left and right M1. Both TMS coils were held tangentially to the scalp, by two different experimenters, with the handle pointing backwards ~45° (ensuring current flow in the brain was in the posterior-anterior direction). The motor “hotspot” of each M1, characterized as the position at which the largest and most consistent MEPs were recorded (Duque et al., 2017), was marked using a felt-tip pen. Both coils could be placed on each M1 without compromising either coil’s positioning relative to the motor hotspot. For each participant’s left and right FDI, the resting motor threshold (rMT)—defined as the lowest stimulation intensity required to evoke motor-evoked potentials (MEPs) of ≥50 μV in three out of five consecutive trials (Hinder et al., 2010, 2011)—was determined at the beginning of the session.

Different dual-coil TMS procedures were administered, both at rest and during different trials of the multicomponent stop-signal task. CSE of pathways to the left and right FDI (CSE_R_ and CSE_L_, respectively) were assessed by delivering dual-coil TMS with an interstimulus interval of 1 ms to the left and right M1 hotspot at 130% rMT of the left and right FDI, respectively (*unconditioned* TMS trials, as the first TMS pulse has no known conditioning effect on the subsequent TMS pulse; Grandjean et al., 2018; Vassiliadis et al., 2018; Wilhelm et al., 2016). This dual-coil procedure allows CSE of both hemispheres to be assessed independently, *but near-simultaneously,* on the same trial. To determine IHI from the right to left M1 (SIHI_RL_ and LIHI_RL_) and from the left to right M1 (SIHI_LR_ and LIHI_LR_), a conditioning TMS pulse at 130% rMT was delivered to the right or left motor hotspot, respectively (Ferbert et al., 1992). SIHI and LIHI were evaluated by delivering the conditioning TMS pulse 10 or 40 ms before the test TMS pulse to the contralateral hemisphere (*conditioned* TMS trials; Chen, 2004; Chen et al., 2003; Ni et al., 2009). SIHI is thought to be mediated via the direct transcallosal inhibitory pathways to the contralateral M1, whereas LIHI is thought to be mediated by indirect pathways, possibly involving premotor regions in both hemispheres (Chen, 2004; Chen et al., 2003; Levin et al., 2014). Regarding the underlying physiological processes, postsynaptic GABA_B_ receptors have been implicated in mediating LIHI (Chen et al., 2003; Irlbacher et al., 2007), although mechanisms mediating SIHI remain unclear. Recent evidence has suggested SIHI and LIHI are independently modulated during movement preparation (Hinder et al., 2018; Puri and Hinder, 2022), providing a strong rationale for assessing both purported pathways in the current study where movement preparation and movement cancellation mechanisms are of interest.

### Data processing

For the behavioural analysis of the stopping interference effect (i.e., stop trial RT relative to the IS), trials with correct responses (go: synchronous bimanual responses; stop: successfully stopping the required hand with the continuing hand recording a RT) and RTs greater than 200 ms (i.e., to exclude “fast guesses” where participants pre-empted the imperative signal or responded to the warning signal) were considered. We also compared successful go response RT with successful stop trials where stop trial RT was considered relative to the stop-signal (e.g., RT relative to the IS) with stop trial RT processed as the difference between the RT (relative to the imperative signal) and SSD.

For the SSRT behavioural analysis, SSRT was estimated nonparametrically for each subject, condition (proactive and reactive), and hand (left and right) based on the independent race model (for a review, see Schall et al., 2017) with all the recommendations adopted from the stop-signal task consensus guide on “when and how to estimate SSRT” (Verbruggen et al., 2019). Specifically, SSRT was estimated using the integration method with replacement of go omissions and was not estimated when a) the probability of responding on stop trials was lower than 0.25 or higher than 0.75 (3 of 108 total estimations based on 27 participants, 2 conditions, and 2 hands), or b) the assumptions of the race model were violated (i.e., mean RT on unsuccessful stop trials > mean RT on go trials; 4 of 108).

For neurophysiological measures, peak-to-peak MEP amplitude was determined in a 10- to 100-ms time window following the test TMS pulse in the left and/or right FDI. Specifically, for CSE trials, MEP amplitude was determined in the left and right FDI, whereas for TMS trials conducted to assess SIHI and LIHI from left to right M1, MEP amplitude was only determined in the left FDI and for TMS trials conducted to assess SIHI and LIHI from right to left M1, MEP amplitude was only determined in the right FDI. In addition, due to the known effects of background EMG activity on MEP amplitude, TMS trials in which root mean square EMG activity exceeded 0.025 mV in a 50-ms time window immediately before the test TMS pulse were excluded from inferential analyses (Puri et al., 2015, 2016).

Lastly, for all behavioural (except SSRT) and neurophysiological measures in the current study, no data aggregation was conducted prior to inferential analyses (i.e., the inferential statistical model was conducted on trial level data). Briefly, even for SIHI and LIHI data, where usually a ratio of average conditioned to unconditioned MEP amplitude is first calculated, the unique feature of the log-link function (i.e., back-transformation of estimates from the link to response scale resulting in ratios; Lo and Andrews, 2015) allowed us to avoid any data aggregation, with ratio values determined using back-transformation (values <1 representing inhibitory interactions and values >1 representing facilitatory interactions; Hinder et al., 2018).

### Statistical procedures

For behavioural measures (“Behavioural effects”), Bayesian generalised linear mixed models (GLMMs) or linear mixed models (LMMs) were utilised. Specifically, separate shifted lognormal GLMMs with an identity link function (Cohen, 2018; De Boeck and Jeon, 2019) were fit with factors of CONDITION (Proactive, Reactive), TRIAL TYPE (Go, Stop), and HAND (Left, Right) for when correct RTs on stop trials was processed relative to the imperative signal (stopping interference effect), as well as when it was processed relative to the stop-signal. Lastly, for averaged SSRT values, a LMM (i.e., Gaussian distribution with an identity link function) was fit with factors of CONDITION (Proactive, Reactive) and HAND (Left, Right).

For movement related neurophysiological measures (“Neurophysiological effects”﻿), Bayesian GLMMs with a Gamma distribution and log link function—appropriate for nonnegative, positively skewed data, such as MEP amplitude—were utilised. To assess movement *preparation* related processing, neurophysiological measures at the beginning (i.e., at WS) and end (i.e., at IS) of the movement preparation period were considered (i.e., before movement execution and cancellation). Specifically, given the lack of foreknowledge in the reactive condition, at IS it was *unknown* whether either hand may be required to stop or not. In contrast, with the foreknowledge provided at the WS in the proactive condition, by the IS it was possible to discern which hand may be required to stop and thus which hand was definitely not stopping (i.e., *definitely going*). Therefore, proactive and reactive conditions are used to form the HAND factor (see below). For example, in the proactive condition, following a “Maybe Stop Left” cue, by the IS it would be known that the left hand may be stopping (thus CSE_R_, SIHI_LR_, and LIHI_LR_ are relevant “maybe stopping” TMS trials) and the right hand would be definitely going (thus, CSE_L_, SIHI_RL_, and LIHI_RL_ are relevant “definitely going” TMS trials), whereas in the reactive condition by the IS it would be unknown which hand may be stopping or definitely going (CSE_R_, SIHI_LR_, LIHI_LR_, CSE_L_, SIHI_RL_, and LIHI_RL_ are relevant “unknown” TMS trials). Accordingly, for CSE, SIHI, and LIHI, separate GLMMs with the factor of HAND (WS, IS_unknown_, IS_maybe_stopping_, and IS_definitely_going_) were fit with the additional factor of TMS TYPE (Unconditioned, Conditioned) for SIHI and LIHI analyses.

To assess movement *execution* and *cancellation* related processing, we analysed the neurophysiological measures at the IS, IS150, and SS150 timepoints for the reactive and proactive conditions (correct responses only). Separate GLMMs (for CSE, SIHI, and LIHI) with factors of HAND (IS, IS150, SS150_continuing_, SS150_stopping_) and CONDITION (Reactive, Proactive) were fit with the additional factor of TMS TYPE (Unconditioned, Conditioned) for SIHI and LIHI analyses. Only contrasts involving HAND are reported, as these effects capture the task-related temporal changes of interest. Specifically, this analysis is focussed on understanding how neurophysiological mechanisms can be regulated over time to execute and cancel movements. Thus, we are primarily interested in contrasts involving HAND and not contrasts solely involving CONDITION.

To determine neurophysiological predictors of stopping success (“Neurophysiological predictors of stopping success”﻿), a Bayesian logistic regression (i.e., Bernoulli distribution with a logit link function) was utilised. Specifically, stopping success (binary outcome) was regressed onto CSE in the “maybe stopping” hand collected at IS and IS150 on proactive stop trials (i.e., stop trials in the proactive condition where prior information about possible stopping demands was provided via the WS).

Following model fitting, main and interaction effects were probed by conducting contrast analyses. Specifically, the posterior distribution of the contrast was utilised to obtain indices of effect *existence* (i.e., the consistency of an effect) and *significance* (i.e., the magnitude of an effect) (Makowski et al., 2019a). For effect existence, the probability of direction (*pd*)—defined as the proportion of the posterior distribution that is of the median’s sign—is reported as a percentage value, varying from 50-100%. The “consistency” of an effect is defined along a continuum between a theoretically most *in*consistent *pd* value of 50%, to a theoretically most consistent *pd* value of 100%. For effect significance, first, a “region of practical equivalence” (ROPE)—a range of values close to zero that are *practically equivalent* to zero (i.e., of negligible magnitude; ±5% for all models except logistic regression where it was set to ±0.05 as per Kruschke, 2018)—was defined. Second, for each contrast, an 89% highest density interval (HDI)—the range within which 89% of the posterior distribution lies—was defined. Then, using the HDI and ROPE, the percentage of HDI falling within the ROPE is reported (*% of HDI in ROPE*). Even though this value is interpretable in itself, the “HDI+ROPE” decision rule (Kruschke, 2018) is also utilised such that a) if the 89% HDI falls completely inside the ROPE, the null hypothesis of no difference (H_0_) is *accepted*, b) if the 89% HDI falls completely outside the ROPE, the null hypothesis is *rejected*, and c) if the 89% HDI does not fall completely inside or outside the ROPE, *no decision* regarding the null hypothesis is reached*.*

Posterior distributions for all models—run using eight independent chains, each with 1,500 warm up and 1,500 post-warm up samples (12,000 total post-warm up samples)—were obtained using the NUTS extension of Hamiltonian Monte Carlo. Improper flat priors over real values were used for population-level parameters. Additionally, the maximal random effect structure (i.e., by-participant random intercepts and by-participant random slopes for all fixed effects as well as correlations among random effects) as allowed by the data and justified by the design was specified for every model (Barr, 2013; Singmann and Kellen, 2019). Convergence of chains was assessed by ensuring that the potential scale reduction factor on split chains (Rhat) was <1.1 (Gelman and Rubin, 1992) and by inspecting plots of post-warm up samples. To ensure models reproduced the observed data, visual posterior predictive checks were conducted to compare the observed and simulated data (Gabry et al., 2019). All graphical outputs and statistical analyses were generated in R version 4.0.2 (R Core Team 2020), via RStudio version 2022.2.0.443 (RStudio Team 2022), using the “here” (Kiril Müller, 2020), “janitor” (Firke, 2021), “tidyverse” (Wickham et al., 2019), “brms” (Bürkner, 2017), “emmeans” (Lenth 2021), “insight” (Lüdecke et al., 2019), “bayestestR” (Makowski, Ben-Shachar, and Lüdecke, 2019b), and “patchwork” (Pedersen 2020) packages with default settings, unless specified above. All data are available via https://osf.io/6bpuh/.

## Results

Table 1 outlines the observed stop-signal task data according to reporting recommendations in the stop-signal task consensus guide (Box 3 in Verbruggen et al., 2019). All model data are reported as medians with the 89% HDI in square brackets, unless specified otherwise.

**Table 1:** **Observed stop-signal task data**. For reactive and proactive conditions, proportions are expressed as percentages and reaction-times are expressed as means in milliseconds with standard deviations.

	Reactive	Proactive
Go omissions	0.2%	0.4%
Go errors (incomplete and asynchronous responses)	6.9%	9.8%
RT on successful go trials	430 ± 89	447 ± 99
Probability of responding on stop trials	0.53	0.53
Average SSD	161 ± 51	163 ± 47
RT on unsuccessful stop trials	400 ± 71	413 ± 79
RT on successful stop trials	533 ± 102	523 ± 108

### Behavioural effects

**Fig. 2:**
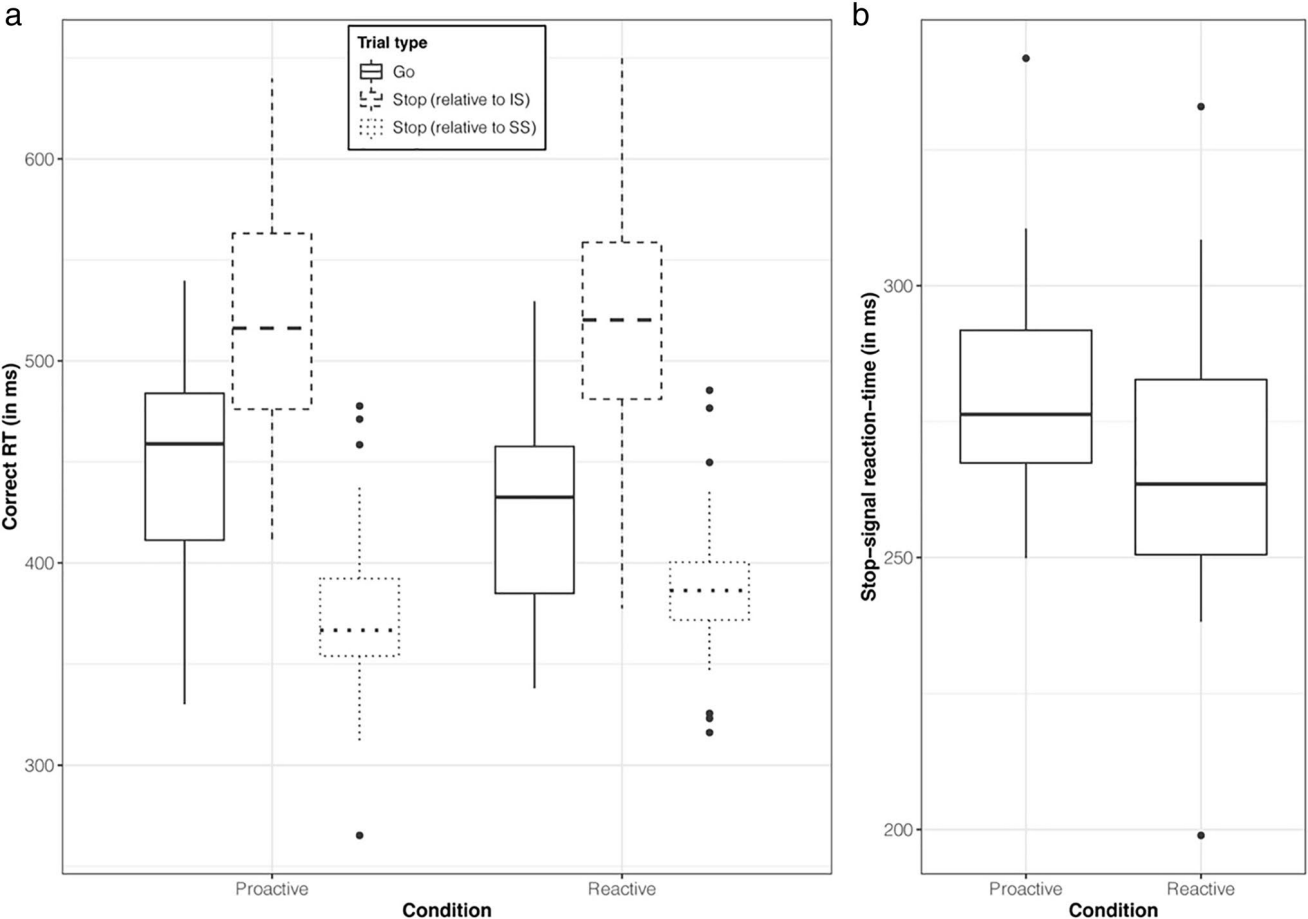
**Behavioural measures. **Boxplots depict, on the ordinate, **a)** the correct RT (in milliseconds) for go trials (solid), stop trials with RT relative to the imperative signal (dashed), and stop trials with RT relative to the stop-signal (dotted), as well as **b)** stop-signal reaction-time, for the proactive and reactive conditions. Lower and upper hinges depict the 25^th^ and 75^th^ percentile respectively with the lower and upper whiskers depicting values 1.5 times the interquartile range (defined as the distance between the 25^th^ and 75^th^ percentile) below and above the hinges, respectively. Data beyond the whiskers are plotted as filled circles

Participants exhibited stopping interference as evidenced by the effect of TRIAL TYPE and illustrated in Fig. 2a (solid and dashed boxplots). Specifically, correct RTs, relative to the imperative go-signal, in the continuing hand on stop trials (518 ms [497–541]) were 19.1% (15.3–22.6) longer than RTs on go trials (435 ms [418–451]), with this effect being both consistent (*pd* = 100%) and significant as the null hypothesis was rejected (0% in ROPE). Furthermore, this effect of TRIAL TYPE was modulated by CONDITION such that stopping interference was 22.5% (13.7–31.5) less in the proactive (73 ms [60–85]) relative to the reactive (94 ms [80–107]) condition, with this effect being both consistent (*pd* = 100%) and significant (0% in ROPE). Thus, the knowledge of possible selective stopping demands in the proactive condition attenuated, but did not eliminate, stopping interference. There was not enough evidence to accept or reject the null hypothesis for the inconsistent three-way interaction between TRIAL TYPE, CONDITION, and HAND (*pd* = 61.4%; 12.3% in ROPE).

Correct RT on successful stop trials in the continuing hand (377 ms [363-392]), *relative to the presentation of the stop-signal*, was 13.6% (9.1–18.0) faster than RT on go trials (436 ms [419–453]) with this effect being both consistent (*pd* = 100%) and significant (0% in ROPE). As illustrated in Fig. 2a (solid and dotted boxplots), this decrease in RT on stop, compared with go, trials was 91.7% (34.3–179) greater in the proactive (78 ms [59–98]) relative to the reactive (41 ms [24–57]) condition, with this effect being both consistent (*pd* = 100%) and significant (0% in ROPE). Although some slowing of go RT was observed in the proactive (445 ms [427–462]) compared with reactive (428 ms [409–446]) condition, the RT of the continuing hand (relative to the presentation of the stop signal, which also acts as the imperative for the selective go response) in successful stop trials was faster in the proactive (367 ms [351–383]) than reactive (387 ms [373–402]) condition. Thus, these analyses unambiguously indicates that proactive cueing results in more efficient (faster) selective responses than in the reactive condition. Lastly, there was not enough evidence to accept or reject the null hypothesis for the inconsistent three-way interaction between TRIAL TYPE, CONDITION, and HAND (*pd* = 56.7%; 19.8% in ROPE).

SSRT, as evidenced by the effect of CONDITION and illustrated in Fig. 2b, was 3.7% (0.9–6.7) greater for the proactive (278 ms [271–285]) than the reactive (268 ms [259–276]) condition. However, although this effect was consistent (*pd* = 99.4%), there was insufficient evidence to determine its practical significance (86.7% in ROPE). In addition, there was not enough evidence to accept or reject the null hypothesis for the inconsistent interaction between CONDITION and HAND (*pd* = 69.2%; 7.0% in ROPE).

Overall, knowledge of selective stopping demands in the proactive condition attenuated, but did not eliminate, the stopping interference effect. The analyses are consistent with the view that the speed of the selective response (relative to the presentation of the stop-signal) is *faster* than the initial bimanual go response (relative to the presentation of the imperative signal) and that this quickening is accentuated by provision of the proactive cue.

### Neurophysiological effects

Due to technical and experimental difficulties, valid TMS data could not be collected on 2.2% (436 out of 19,710) of TMS trials. Resting motor thresholds were similar between the left (mean = 36.5% of maximum stimulator output; SD = 5.7%) and right (mean = 36.1% of maximum stimulator output; SD = 5.4%) hemispheres.

#### Movement preparation

**Fig. 3:**
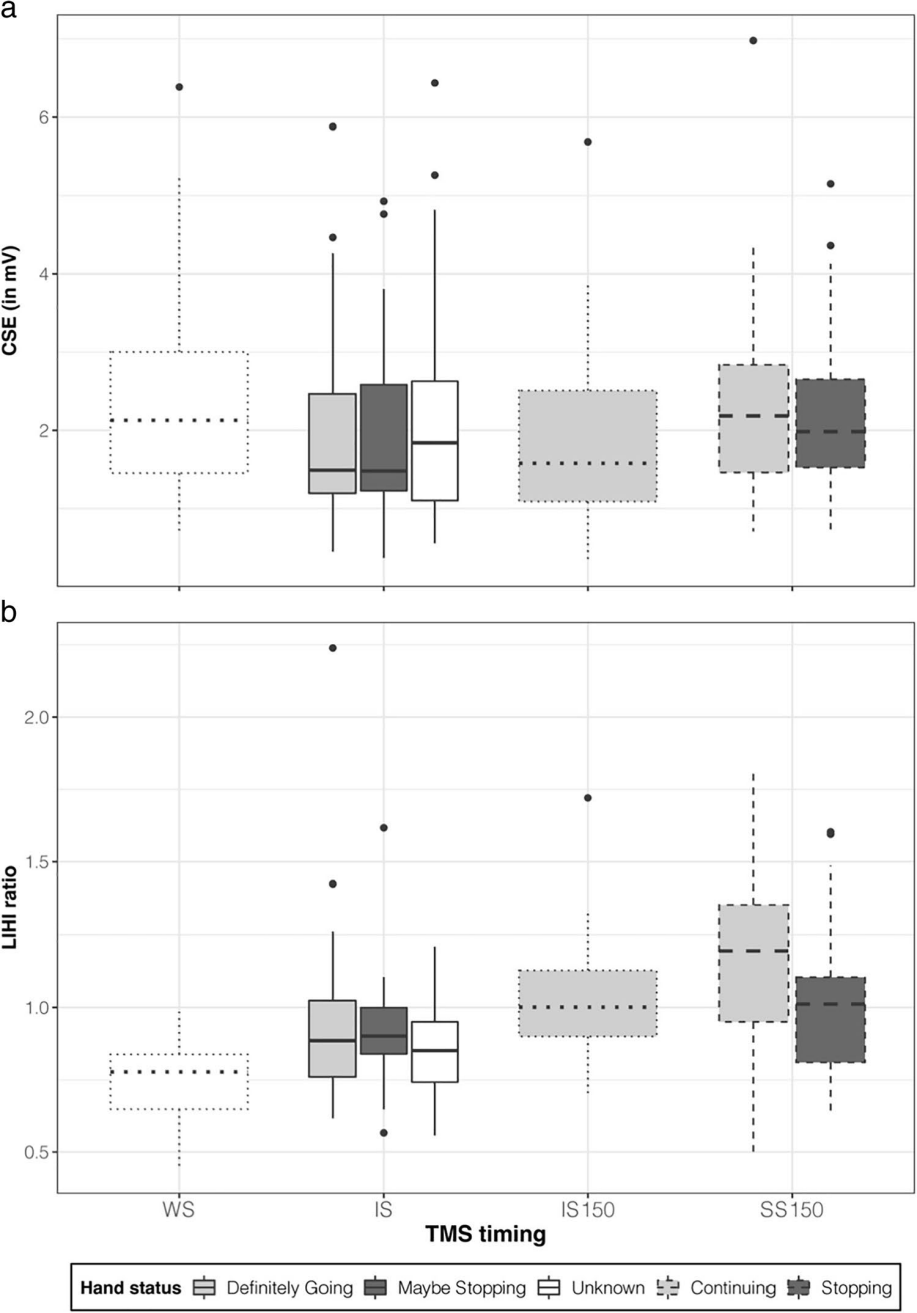
**Neurophysiological measures.** Boxplots depict, on the ordinate, **a)** CSE (in mV), and **b)** LIHI ratio, at the warning signal (WS), imperative signal (IS), 150 ms after the IS (IS150) and stop-signal (SS150) time-points on the abscissa. These are plotted for various hand statuses, specifically at WS when there is no relevant foreknowledge (unfilled dotted), at IS (proactive condition leading to *definitely going*—light grey solid—and *maybe stopping*—dark grey solid, and reactive condition leading to *unknown*—unfilled solid), at IS150 when the imperativeness of the go-signal dominates (light grey dotted), and at SS150 (*continuing hand*: light grey dashed; *stopping hand*: dark grey dashed). LIHI ratios less than and greater than 1 represent inhibitory and facilitatory interactions, respectively. Lower and upper hinges depict the 25^th^ and 75^th^ percentile respectively with the lower and upper whiskers depicting values 1.5 times the interquartile range (defined as the distance between the 25^th^ and 75^th^ percentile) below and above the hinges, respectively. Data beyond the whiskers are plotted as filled circles

For CSE, in the proactive condition, consistent (all *pd*s = 100%) and significant (all 0% in ROPE) reductions in CSE were observed at IS relative to the WS. These reductions at IS were observed regardless of whether the hand was definitely going or maybe stopping (IS_definitely_going_ and IS_maybe_stopping_ in Fig. 3a). Specifically, CSE was 23.6% [15.1–31.7] and 24.3% [15.8 – 32.2] lower in the hand that was definitely going (1.65 mV [1.29–1.98]) and maybe stopping (1.63 mV [1.29–1.97]) compared with CSE at WS (2.15 mV [1.73–2.59]). Furthermore, at IS, CSE in both hands during the proactive condition were consistently (definitely going: *pd* = 99.2%; maybe stopping: *pd* = 99.3%) lower than that observed in the reactive condition at IS (1.86 mV [1.48–2.28]; IS_unknown_ in Fig. 3a), although these were not of sufficient practical significance (definitely going: 4.2% in ROPE; maybe stopping: 2.8% in ROPE). Similarly, though the reductions observed in the reactive condition at IS were consistently (*pd* = 99.2%) lower than that observed at WS, they were also not of sufficient practical significance (2.9% in ROPE).

For LIHI, consistent (all *pd*s = 100%) and significant (all 0% in ROPE) reductions in inhibition were observed at IS during the proactive condition in the hand definitely going (0.92 [0.85 – 1.00]; IS_definitely_going_ in Fig. 3b) and maybe stopping (0.90 [0.83–0.98]; IS_maybe_stopping_ in Fig. 3b) compared with LIHI at WS (0.73 [0.69–0.78]). For all other contrasts, there was not enough evidence to accept or reject the null hypothesis for any contrast (*pd*s < 99.1% with 4.9–57.8% in ROPE).

For SIHI, though SIHI was captured adequately at the WS as evidenced by a SIHI ratio of 0.69 [0.64–0.75], there was not enough evidence to accept or reject the null hypothesis for any contrast (*pd*s < 99.0% with 5.6–56.9% in ROPE).

Overall, at the end of the movement preparation period (at IS), CSE decreases as well as reductions in the extent of LIHI were observed during the proactive condition regardless of whether foreknowledge indicated the hand was definitely going or maybe stopping, suggesting generic (across both hands) neurophysiological mechanisms related to processing of the available foreknowledge. We propose that changes to LIHI and CSE are occurring as independent mechanisms, as reductions in indirect interhemispheric inhibition would otherwise be expected to *increase* CSE. Conceivably, it may be that other mechanisms, such as increased intracortical inhibition and increased transcortical inhibition from other brain regions, may be playing a role, exclusively or concurrently, to mediate the observed CSE reductions.

#### Movement execution and cancellation

For CSE, consistently (all *pd*s > 99.3%) and significantly (all < 2.0% in ROPE) greater CSE was observed at SS150 for both the continuing hand (2.13 mV [1.74–2.56]; SS150_continuing_ in Fig. 3a) as well as the stopping hand (2.01 mV [1.64–2.40]; SS150_stopping_ in Fig. 3a) compared with CSE at IS (1.74 mv [1.40–2.10]) and at IS150 (1.61 mV [1.29–1.96]). Specifically, CSE at SS150 in the continuing hand was 22.4% [7.5–37.5] and 32.2% [15.3–49.0] greater than CSE at IS and IS150, respectively. For the stopping hand, CSE at SS150 was 15.6% [3.5–29.1] and 24.8% [11.3–39.7] greater than CSE at IS and IS150, respectively. For all other contrast analyses involving HAND, there was not enough evidence to accept or reject the null hypothesis (*pd*s < 98.3% with 21.9–59.5% in ROPE).

For LIHI, a consistent (*pd* = 99.8%) and relatively significant (1.2% in ROPE) 13.9% (4.2–23.9) reduction in inhibition was observed at IS150 (0.99 [0.92–1.06]) compared with IS (0.87 [0.81–0.93]). In addition, of relevance to the current study, a reduction in LIHI was observed at SS150 in the continuing hand (SS150_continuing_ in Fig. 3b). Specifically, there was a 28.8% [12.5–45.9] reduction in LIHI at SS150 in the continuing hand (1.12 [1.01–1.24]) compared with IS (*pd* = 100%; 0% in ROPE). This LIHI reduction was 13.1% greater [−0.02–28.8] than LIHI observed at IS150 and though it was relatively consistent (*pd* = 96.3%), it was not practically significant (12.9% in ROPE). Furthermore, the LIHI reduction in the continuing hand was 15.1% greater [−1.9–34.3] than LIHI observed in the stopping hand at SS150 (0.97 [0.88–1.06]; SS150_stopping_ in Fig. 3b), and although this was relatively consistent (*pd* = 95.9%), it was not of sufficient practical significance (11.2% in ROPE). For all other contrast analyses involving HAND, there was not enough evidence to accept or reject the null hypothesis (*pd*s < 96.9% with 11.1–59.9% in ROPE).

For SIHI, there was not enough evidence to accept or reject the null hypothesis for any contrast involving HAND (*pd*s < 99.5% with 3.2–57.4% in ROPE).

Overall, 150 ms after the stop-signal, nonspecific CSE increases were observed in both the continuing and stopping hand. However, a significant hand-specific LIHI reduction was observed in the continuing hand 150 ms after the stop-signal to possibly assist movement execution in that hand.

### Neurophysiological predictors of stopping success

Overall, there was not enough evidence to accept or reject the null hypotheses for CSE in the maybe stopping hand at IS (slope: 0.03 [−0.07—0.13]; *pd* = 73.9%; 62.3 % in ROPE) or IS150 (slope: −0.02 [−0.21—0.16]; *pd* = 59.4%; 44.4 % in ROPE) predicting stopping success.

## Discussion

The current study provides novel behavioural and neurophysiological insights into reactive and proactive response-selective stopping in healthy young adults. From a behavioural perspective, contextual cues, termed foreknowledge, about potential stopping in one limb reduced stopping interference in the continuing limb. Moreover, the speed of the continuing response (relative to the stop-signal) in successful selective stop trials was faster than the bimanual response (relative to the imperative signal), with this speeding of response accentuated by the provision of a proactive cue. From a neurophysiological perspective, the significant reduction in LIHI observed 150 ms after the stop-signal in the continuing limb highlights the pivotal role played by interhemispheric inhibitory connections between contralateral primary motor regions, mediated via prefrontal regions, in response-selective stopping.

### Behavioural effects

In line with previous literature, we observed stopping interference (~100 ms) in the reactive condition that was attenuated (~20%) by providing participants foreknowledge of upcoming stopping demands (Aron and Verbruggen, 2008; Claffey et al., 2010; Lavallee et al., 2014; Raud and Huster, 2017; Smittenaar et al., 2013). However, if we conceptualise the stop-signal on selective stop trials to serve not only as a stop-signal for the planned bimanual movement but also as an *imperative* signal for the continuing effector to initiate a unimanual (selective) action, a different pattern of results is observed. Specifically, RT of the continuing hand in successful stop trials is *quicker*, relative to the bimanual go RT, in the reactive condition (~40 ms) with this quickening almost doubled for the proactive condition (Fig. 2). Both of these behavioural effects suggest that participants effectively utilize information about response-selective stopping demands to quicken the speed of the continuing effector. One possibility of how this may occur could be understood in the context of ‘coupling’ between subcomponents of a multicomponent response (for a review, see Shea et al., 2015). In the current study, given the predominant response is the bimanual go response (2/3^rd^ of all trials), the left and right index fingers are likely to be functionally coupled to reduce processing costs (Wenderoth et al., 2009). However, the functional coupling aiding efficient bimanual go responding is detrimental to the demands of response-selective stopping as it initially requires an uncoupling of the subcomponents before one can be stopped and the other continued. By providing foreknowledge of stopping demands in the proactive condition, it is possible that functional coupling between effectors could be weakened to aid successful stopping of one effector and quicker continuation of the other effector. Based on this hypothesized weakened functional coupling in the proactive condition, one would expect not only quicker selective responses (i.e., reduced stopping interference) but also slower and more variable bimanual go responses compared with the reactive condition. Indeed, stopping interference is reduced in the proactive, rather than reactive, condition, but bimanual go RT is longer and more variable (Table 1 and solid boxplots in Fig. 2a). Moreover, stopping interference is reduced in other experimental manipulations that weaken the degree of functional coupling between effectors, such as when they are paired heterogeneously (MacDonald et al., 2012) or require an asynchronous go response (Wadsley et al., 2019).

This quickening of the continuing effector in the proactive condition appears to occur in conjunction with a consistent, but not practically significant, slower stop latency (SSRT) of the stopping effector (Fig. 2b). Our finding of somewhat increased SSRT in the proactive, compared to reactive, condition has been reported in some previous studies (Aron and Verbruggen, 2008; Claffey et al., 2010 Experiment 1), and given the increased activation of the striatum in a proactive response-selective context (Majid et al., 2013), could suggest a possible role of the slower indirect, rather than hyperdirect, pathway in proactive response-selective stopping. However, studies have also found no significant difference in SSRT between proactive and reactive conditions (Raud and Huster, 2017; Claffey et al., 2010 Experiment 2) and given the high HDI percentage in ROPE (~87%) in the current study, alternative viewpoints are likely. Indeed, it may be that proactive cueing has more influence on the speed of the go processes (i.e., slower bimanual go response and quicker continuing response), with little to no influence on the speed of the stop process (i.e., SSRT), which occurs via a fast hyperdirect pathway, regardless of cueing context. Finally, we note that *decreased* SSRT in the proactive (compared to reactive) condition has also been reported (Smittenaar et al., 2013); thus, the inconsistency in cue-related SSRT changes in response-selective stopping tasks may question the underlying assumptions of SSRT estimation in a response-selective context (Bissett et al., 2021; Matzke et al., 2017), rather than being indicative of changes in underlying mechanisms, per se.

### Neurophysiological effects

In the movement preparation period (i.e., before IS processing), reductions in CSE were observed in the reactive and proactive conditions (in both the hand that was definitely going and maybe stopping). One may expect, if CSE were to reflect response-selective preparatory mechanisms, that CSE would be lower in the hand that maybe stopping (and higher in the hand that was definitely going). However, it is likely, given that stop trials only constituted of one third of trials in the task, that neural mechanisms in the preparatory period were driven by demands of the more frequent bimanual go response (2/3^rd^ of all trials). In that regard, CSE reductions in the preparatory period of response execution tasks, appropriately termed “preparatory inhibition,” are a robust finding and are best explained in the context of the “inhibition for impulse control” hypothesis (for a review, see Duque et al., 2017). Specifically, the widespread reductions serve to prevent the release of action representations, as evidence is gathered to select the most appropriate representation, until an imperative cue dictates the initiation of the selected action. Moreover, given that there was more opportunity to prepare in the proactive, than reactive, condition by way of the informative warning cue (i.e., the cue alerted the participant to suppress not only the bimanual go response, but also the response of the hand that would be definitely going), it is perhaps unsurprising that consistently, but not practically significant, greater CSE reductions were observed in the proactive, than reactive, condition.

In addition, significant LIHI reductions were observed in the movement preparation period but only in the proactive condition. Provision of task-relevant information (pertaining to the stopping demands of the task) in the proactive condition during the preparatory period is likely to have made the proactive condition more cognitively demanding compared to the reactive condition where no information was provided. As such, LIHI networks that are thought to be mediated by more (pre)frontal regions and influenced by top-down processes (Hinder et al., 2018; Ni et al., 2009; Puri and Hinder 2022) may have played a more vital role in the proactive condition with reductions possibly subserving the more frequent bimanual go response.

In the movement cancellation period (i.e., 150 ms after the SS), CSE increases were observed in both the continuing *and* stopping hand. In the continuing hand, a CSE increase seems plausible (to potentially assist response continuation), but a CSE increase in the stopping hand is surprising even though numerical increases have been reported in the literature (from 175 to 225 ms post SS as per Figure 6 in MacDonald et al., 2014; from 175 to 200 ms post SS as per Figure 2b in Cowie et al., 2016; at 150 ms post SS as per Figure 4a in Raud et al., 2020). Given that MEP amplitude from unconditioned TMS is a summed output of direct and indirect (via intra-, inter-, and sub-cortical inputs) activation of corticospinal neurons (Bestmann and Krakauer, 2015), no specific inferences can be made in regard to the CSE increase in the stopping hand. Speculatively, in the context of the coupling hypothesis, it may be that CSE increases 150 ms post SS reflect an increased motor drive to aid “decoupling” of the two subcomponents, resulting in the continuation of the required effector at an average of ~370 ms post SS (and possible CSE increases around that timepoint) and stopping of the other effector at an average of ~270 ms post SS (and possible CSE decreases around that timepoint) as illustrated in Fig. 2.

The key neurophysiological finding in the current study provides novel insights into the role of LIHI networks in subserving the demands of response-selective paradigms. Specifically, a significant reduction in LIHI was observed in the continuing hand 150 ms after the SS compared with LIHI at IS (Fig. 3b). Moreover, this LIHI reduction in the continuing hand was consistently, but not practically significant, greater than that observed in the stopping hand, measured at the same timepoint, as well as both hands in the bimanual go response, measured 150 ms after the IS. The significant LIHI reduction in the continuing hand can be best understood within the “activation-threshold” framework (MacDonald et al., 2017). In this framework, the response threshold for an effector is elevated after the stop-signal due to the recruitment of inhibitory neurophysiological processes. Thus, an *additional* phase of physiological facilitation is required to reach this elevated threshold and enable a rapid response of the continuing effector. Indeed, EMG recorded from the continuing effector following the stop-signal is observed at a greater gain than that for a comparable go response (for a review, see Wadsley et al., 2022). Given that response-selective stopping is a complex form of response inhibition, LIHI networks, that are thought to be mediated by more (pre)frontal regions and influenced by top-down processes (e.g., SIHI which represents direct transcallosal connections between contralateral M1s without influence of frontal brain activity) are likely to have played a fundamental cortical role in reaching this elevated threshold and assisting the response of the continuing effector.

### Future directions and conclusions

Future studies may build upon the insights of the current research in various ways, some of which are outlined here. First, the neurophysiological data in the current study did not provide evidence to accept or reject the null hypothesis for any SIHI related effects and some foreknowledge effects. To this end, a larger sample size and/or more trials for the various conditions may prove beneficial. Second, it would be beneficial to administer TMS at multiple timepoints to gain a deeper understanding of the temporal changes in inhibition and excitation, particularly at the same timepoint following the IS on both go and stop trials. Third, given that projections suggest that the number of people aged 65 years and older will double in the next three decades (United Nations Department of Economic and Social Affairs, 2020), it is paramount to further our understanding of selective stopping in this cohort. Indeed, research has shown age-related effects of foreknowledge as well as GABA levels in key nodes of the cortico-subcortical pathways subserving response inhibition (Pauwels et al., 2019). Similarly, future research could elucidate mechanisms in clinical groups exhibiting movement disorder symptoms, such as tics, stuttering, and freezing of gait (Hannah and Aron, 2021). Fourth, the overlap (or lack of thereof) between non-, stimulus-, and response-selective stopping in experimental and real-world settings remains poorly understood and studies could use within-subject comparisons to deepen our understanding.

In conclusion, the current study investigated the behavioural and neurophysiological underpinnings of proactive and reactive response-selective stopping. Behaviourally, stopping interference was reduced and the speed of the selective response was quicker in the proactive, compared with reactive, condition. On a neurophysiological level, we provide the first evidence of the role of indirect interhemispheric inhibitory connections between contralateral M1s, mediated via prefrontal regions, in a response-selective stopping paradigm.

## Data Availability

All data are available via https://osf.io/6bpuh/.

## Footnotes

1 Thereafter, in the current study, experimental conditions where no or some foreknowledge of stopping demands are provided are referred to as “reactive” and “proactive,” respectively.

## Code availability

Code can be made available upon request.
